# Moving towards chemical-free agriculture, 37 kb at a time

**DOI:** 10.1093/synbio/ysab009

**Published:** 2021-02-15

**Authors:** Sonja Billerbeck

**Affiliations:** Department of Molecular Microbiology, Groningen Biomolecular Sciences and Biotechnology Institute, University of Groningen, Groningen, The Netherlands

Domestic crop plants are modern marvels of extensive breeding; however, many of their natural defenses against pests and pathogens have been lost. Wild relatives still harbor disease resistance genes, but transferring these large sequences into complex, polyploid plant genomes calls for advanced genomic engineering technologies. Recently, government researchers in Australia, successfully transferred a 37 kb resistance stack into the genome of a domesticated wheat species such that it is protected against the rapidly evolving wheat leaf rust pathogen *Puccinia graminis f.* sp. *tritici* (*Pgt*) without losing any agronomic features.^1^

Plant diseases caused by pathogenic fungi can devastate crop yield and pose a threat to food security.^2^^,^^3^ About 30% of our most important crops are lost every year to fungal diseases.^3^ Over decades, agricultural crops have been bred towards maximum productivity under high fungicide treatment, meanwhile breeding out the plants’ own defense genes.^3^ The genetic armory still intact in wild crop relatives (so-called R genes) could provide an effective means towards a chemical-free disease control.^4^ Introducing those genes into domestic crops is a multifactorial challenge yet underappreciated by much of the synthetic biology community.

While most microbially focused synthetic biologists routinely move multiple genes and whole pathways from one organism to another, the genetic engineering of single R genes into wheat is still considered very difficult. With an average size of 8 kb, R genes are very large, and genetic manipulation of wheat is challenged by its large complex polyploid genome.^5^

Further, introducing a single R gene into a crop might not provide sufficient armory: First, pathogenic fungi have the capacity to quickly evolve to overcome plant resistance after a few seasons. Second, different geographical isolates of pathogenic fungi show various types of resistance and those isolates can rapidly spread globally due to human activity.

Last month, a research team lead by Michael Ayliffe provided a potential solution to the crop-disease challenge by developing a modular, broad-spectrum resistance engineering approach, reported in *Nature Biotechnology*.^1^ The researchers combined five R genes belonging to two mechanistically different resistance classes into a so-called ‘resistance stack’, integrated into a single locus of the wheat genome. Several of these genes had been isolated by the researcher’s collaborators at the John Innes Center (UK) by using a new rapid R-gene-identification technique called MutRenSeq.^2^^,^^5^

The presented engineering approach solved two design challenges: first, all R genes need to be stacked into a single genomic locus, such they can be stably inherited all together. Second, the insertion strategy needs to be modular, such that R genes can be replaced by others to create custom resistance profiles in order to strategically engineer plants for geographical needs.

Therefore, the team developed a new restriction enzyme-free reiterative gateway cloning strategy in *Escherichia coli*, suitable to clone the extremely large resistance stack (37 kb all together) into a multi-transgene cassette. The cassette was then transferred into the wheat cells via a new high-efficiency Agrobacterium-based wheat transformation system—an engineered version of the natural way that the bacterial plant pathogen *Agrobacterium tumefaciens* uses to inject its DNA into plant cells.

The researchers then showed that three out of 80 transformed plants (∼27%) carried the correct five-gene insertion at the correct locus and that this engineered locus could be stably inherited. Using a collection of seven geographically distinct *Pgt* isolates with different virulence profiles, they demonstrated that the multi-transgene cultivars showed broad-spectrum resistance with no measurable agronomic phenotypic detriment in field studies.

As such several questions remain: how scalable is the method? That is, how many more R genes can be added per stack and can several stacks be combined? Further, how many mechanistically different R genes are encoded in wild genomes? And can rapid R gene identification via MutRenSeq, combined with the herein featured technology, provide the necessary pace to enter the evolutionary arms race between crops and fungal pathogens?

Alarmingly—during the study—a new, highly virulent *Pgt* isolate appeared in Sicily^6^ that showed resistance against three out of the five engineered R genes. Clearly, R gene transfer alone, even when accomplished at scale will be insufficient to compete with fungal evolution. This dilemma bids the question—could direct protein evolution be harnessed to expand the variety and potency of R genes?^7^ Perhaps, this will be the subject of the next great paper by the small and growing community of plant synthetic biologists.
